# Mapping the Complex Morphology of Cell Interactions with Nanowire Substrates Using FIB-SEM

**DOI:** 10.1371/journal.pone.0053307

**Published:** 2013-01-09

**Authors:** Rafał Wierzbicki, Carsten Købler, Mikkel R. B. Jensen, Joanna Łopacińska, Michael S. Schmidt, Maciej Skolimowski, Fabien Abeille, Klaus Qvortrup, Kristian Mølhave

**Affiliations:** 1 DTU Nanotech, Technical University of Denmark, Lyngby, Denmark; 2 DTU CEN, Technical University of Denmark, Lyngby, Denmark; 3 Department of Biomedical Sciences, CFIM, University of Copenhagen, Copenhagen, Denmark; Argonne National Laboratory, United States of America

## Abstract

Using high resolution focused ion beam scanning electron microscopy (FIB-SEM) we study the details of cell-nanostructure interactions using serial block face imaging. 3T3 Fibroblast cellular monolayers are cultured on flat glass as a control surface and on two types of nanostructured scaffold substrates made from silicon black (Nanograss) with low- and high nanowire density. After culturing for 72 hours the cells were fixed, heavy metal stained, embedded in resin, and processed with FIB-SEM block face imaging without removing the substrate. The sample preparation procedure, image acquisition and image post-processing were specifically optimised for cellular monolayers cultured on nanostructured substrates. Cells display a wide range of interactions with the nanostructures depending on the surface morphology, but also greatly varying from one cell to another on the same substrate, illustrating a wide phenotypic variability. Depending on the substrate and cell, we observe that cells could for instance: break the nanowires and engulf them, flatten the nanowires or simply reside on top of them. Given the complexity of interactions, we have categorised our observations and created an overview map. The results demonstrate that detailed nanoscale resolution images are required to begin understanding the wide variety of individual cells’ interactions with a structured substrate. The map will provide a framework for light microscopy studies of such interactions indicating what modes of interactions must be considered.

## Introduction

Nano- and micro-fabricated structured substrates achieve an increasing amount of interest in cell biology, where their uses are as diverse as biochemical manipulation [1], [2], supporting and controlling cell movement [3]–[5], electrophysiological measurements [6]–[8] and intracellular measurements [9], [10]. Despite this multitude of uses and large interest in nanowires in cell biology, the basic modes of interaction between nanostructured substrates and cells are poorly understood, both in terms of the topography on an ultrastructural level, and in terms of the biological processes when compared to for instance endocytosis of dispersed particles [11], [12] where several pathways have been studied intensely.

Examples in literature often show images of critically point dried (CPD) cells imaged by a scanning electron microscope (SEM). This method provides excellent images showing how cells lie on the particular substrate, and one can get an idea of the level of interaction with the substrate by cell protrusions such as lamellipodia [2], [4], [10], [13], [14]. However it cannot be seen how the nanowires behave below or inside the cells. Combining CPD cells on substrates and focused ion beam SEM (FIB-SEM) does provide some answers about the cell-substrate interaction, but CPD leaves little intracellular ultrastructure intact [15]–[17]. Drobne *et al*. managed to obtain some detail by critically point drying a chemically fixed and stained digestive gland epithelium and demonstrates that FIB-SEM can be used for imaging internal structures in biological samples [18]. The method proved suitable for obtaining gross tissue morphology and comparison with embedded TEM images, but the method lacks intracellular detail due to poor contrast which is also illustrated in [3].

Studies have also been done with light microscopy methods such as confocal microscopy, were cells have been imaged in contact with nanostructures in the form of substrates or probes [2], [10], [19]. These images can be made in physiological relevant solutions, but they require fluorescent labelling and are generally resolution limited to about 200 nm [20].

Transmission electron microscopes (TEM) together with heavy metal stained and embedded samples provide high resolution and detailed ultrastructural information in biological specimens [3], [8], [21], [22]. The required thin samples are typically cut by an ultramicrotome. However, for composite samples also containing glass or silicon substrates as used in this work, there is a risk of delamination and distortions during ultramicrotomy [23], [24]. Therefore the substrate is often removed prior to thin sectioning by either etching [8], [22], temperature induced cleavage [3], [15] or other methods [21], [25], [26]. Exceptions are Dalby *et al*., who manages to avoid substrate removal as they use PMMA structured substrates which can be sectioned by an ultramicrotome [27], and Gnauck *et al*., who uses a FIB to gain access to fibroblast cells on silicon microstructures [28]. Substrate removal could pose an obstacle if nanostructured substrates are to be removed mechanically as the process risks deformation of the nanostructures, but if suitable chemical agents exist, part of or all the substrate can be chemically etched away leaving the structures intact [8]. By using FIB-SEM substrate removal is not required and this is beneficial in circumstances when the substrate for some reason cannot be removed and is not suitable for microtomy. Alternatively, one could make use of lamella cut-outs made using a focused ion beam (FIB) and image them in the TEM, but this is a very time consuming process [29] although providing higher ultimate resolution than SEM. Here we use block face imaging with the FIB-SEM to image multiple-cell volumes at the expense of the higher resolution in TEM.

The large interest in nanostructures and their possible applications in cell biology have sparked many studies investigating the cell-substrate interactions. In 2004, Dalby *et al*., published a study showing how fibroblast would use filopodia to probe a substrate covered with PMMA nanopillars. They provided SEM images of CPD cells and TEM images of embedded cells [27]. Several other studies have also been published on the subjects of cell morphology [27], [30], differentiation [31]–[33], and motility [4], [34] on nanostructured substrates. There is in particular a large interest in excitable cells on nanostructures for electrical signalling and recording. For instance increasing cell signalling by growing cells on CNT covered substrates [7], [35], or close-proximity or penetrating nanostructured arrays for measurement and activation [2], [8], [36], [37]. Several electron microscopy studies have been made of the interfaces [8], [15], [16]. In the Thomson Reuters Web of Science database, the search term “nanowire* and cell* and bio*” indicates about 200 publications per year in the field. It highlights the importance of furthering our knowledge of cell-nanostructure interactions, and the need for categorising the effects we see to gain an insight into the biology involved as has been partly done with endocytosis of nanoparticles [11], [12].

*In-situ* FIB-SEM imaging gives the opportunity to do serial block face imaging which can be reconstructed to a 3D representation of the sample and provide a large 3D image volume [38]. Several reports present how FIB-SEM can be used to image frozen biological samples [24], [29], [39], but ultrastructure visibility is limited due to the poor contrast. Combining the techniques known from polymer embedded TEM samples, and the fast FIB-SEM method it is possible to achieve a fair quality of the ultrastructure and volume [3], [21], [26], [38]–. Except for Bittermann *et al*., the literature on embedded FIB-SEM on biological samples tends to focus on various forms of substrate removal as was the case for TEM – depending on the sample this may introduce artefacts or simply be impractical. The focus on removal comes from the embedding method which leaves a large volume of resin above the cells, and removing the substrate makes the cells easily available from below.

In this paper we present a study of block face FIB-SEM imaging of polymer embedded 3T3 Fibroblast cell monolayers on nanostructured substrates without prior removal of the substrate. To our knowledge we are one of the few (apart from [28]) to show FIB-SEM images of resin embedded cells on nanostructured samples without any removal of the underlying substrate, and present FIB-SEM images of cells cultured on a set of different substrates: Flat glass is used as a reference and two morphologies of silicon nanowires are used. We tested both a tilted- and a non-tilted milling approach depending on the sample.

We present an overview map of the observed interactions between the nanostructured substrate and the cells. Some of these intricate interactions have, to our knowledge, not been reported previously and demonstrate how complex these can be. For example we observed how nanowires were broken off from the substrate and subsequently engulfed by the cells and ordered in tightly packed clusters. We also observed how microvilli of cells could probe into the nanostructures they rested on. Lastly we also show an instance of nanowires indenting the nucleus without penetrating it. This leads to numerous issues to consider when performing light microscopy on such samples as many of the nanostructures used for optical studies are often not directly observed by e.g. fluorescence from the nanostructure itself. The map also provides a starting point for organizing observations from the many different reported experiments and is a beginning to categorise the many different interactions and eventually studying the detailed underlying pathways.

## Materials and Methods

### Nanostructure Substrate Fabrication

Two different black silicon substrates also known as “nanograss” [41] were used: one provides high density silicon nanograss (Nanograss A), while the other has sparser nanowires (Nanograss B). A table of the substrates’ different characteristics can be seen in Table 1, refer to Figure S1 for SEM images of the substrates.

**Table 1:** Overview of the different nanostructured substrates, their processing parameters, and their morphology.

Sample	Height [nm]	Width [nm]	Density [1/µm2]
Nanograss A	990±190	80±60	9.6±0.8
Nanograss B	1170±280	70±40	4.5±0.3

The uncertainties are 2 times the standard deviation giving a two sigma/95% confidence.

The black silicon nanograss was made from 4″ low doped silicon wafers using maskless deep reactive ion etching (DRIE). Differing nanostructures were obtained by controlling the reactive ion etch parameters [42]. For instance the density is controlled by varying the process chamber pressure and coil electrode power, whereas the height scales linearly with processing time. DRIE was performed in an advanced silicon etcher (Surface Technology Systems), the SF_6_/O_2_ ratio was 1.11, while the platen power was 120 W, and the chamber pressure was between 8 and 56 mTorr. This formed nanostructured “silicon grass” at a rate of about 2 nm/s [42].

### Cell Monolayer Culturing

Mouse embryonic fibroblasts (NIH3T3) were cultured on plain glass substrates, and 10×10 mm diced silicon chips with Nanograss A and Nanograss B (Table 1). Before culturing, the chips were sterilised with 70% ethanol for 20 minutes, and flushed 3–4 times with pure water or PBS. The cells were cultured in Dulbecco’s modified Eagle’s medium with Glutamax (DMEM; GIBCO Life Technologies), 10% fetal bovine serum (FBS; Sigma) and 1% penicillin-streptomycin (P/S; GIBCO Life Technologies). Standard conditions of 37°C and an atmosphere of 5% CO_2_ were applied. As capillary forces during drying is known to incur nanowire bending and clustering [43], care was taken to always have liquid covering the samples during preparation.

### Cell Monolayer Post-culture Processing

After culturing for 72 hours the cells were fixed, stained and embedded (cf. Text S1 for the full protocol). First, the samples were fixed with 2% glutaraldehyde in 0.05 M sodium cacodylate buffer, pH 7.2 (isotonic, 300 mOsm) for 1 hr, rinsed in 0.15 M sodium cacodylate buffer pH 7.2 (2×30 min), and postfixed in 1% osmium tetroxide in 0.12 M cacodylate buffer pH 7.2 (isotonic, 300 mOsm) for 1 hour. Next, the specimens were rinsed in Milli-Q water (2×10 min) to remove osmium residues, and stained with 1% tannic acid in Milli-Q for 1 hr. Following a rinse in Milli-Q (2×10 min), the sample was stained with 1% uranyl acetate for 2 hrs. The specimens were dehydrated and embedded in Epon according to standard procedures, please refer to Text S1 for the full protocol.

The polymerised Epon formed a meniscus over the substrate, leading to a thick resin layer in the centre and a thinner layer near the chip edges. This meant that a circular band of cells were directly accessible with the FIB-SEM, with an excessive thick layer in the centre which thinned out towards the periphery leaving only collapsed cells outermost.

### FIB-SEM

Two FIB-SEM beam systems from FEI were used: the Quanta FEG 3D, and the Helios NanoLab600. The first system makes use of a dedicated backscatter detector and the second an in-lens detector.

The cells of interest were localised from atop in standard SEM, using the highest acceleration voltage (30 kV) to detect cells underneath the embedding material (cf. Figure S2). In this paper, results are presented which were typically buried 5 µm deep in the embedding medium (cell top to surface). When a cell of interest was located, the acceleration voltage was lowered to 1.5–5 kV depending on the equipment and crossover alignment of both electron and ion beams was performed. To gain access to the cell, rough milling at high ion beam current was used, forming a trench in front of the cell. The time for trench milling was approximately 10–20 minutes, followed by finer milling prior to image recording.

Both microscopes have installed *G2 Slice and View* software provided by FEI Company. It offers recording of slice stacks with a practical slice thickness as low as 10 nm in our experience, and image sizes and resolution allowing detailed imaging of whole cells. The thickness is limited by the ion beam alignment and stability and not the software. Automatic refocusing of the image is possible when the specimen holder is tilted and milling is done normal to the sample surface, but not for larger samples where non-tilted milling had to be performed (also called slanted milling [40]).

To avoid damaging the dedicated vC backscatter detector in the Quanta FEG 3D large samples could not be tilted. Thus to compare non-tilted and tilted sample images a post-processing algorithm was developed to get representative image volumes and comparable images (please refer to Text S2). Besides allowing milling of large samples, another advantage of non-tilted milling is the decreased brightness gradient resulting from deep trench imaging [40]. However, this process is more computational heavy, and suffers more if the slice thickness is not sufficient for resolving 1D nanostructures compared to tilted-milling.

If the slice thickness is not sufficiently small for resolving the 1D nanostructures, slanted milling (horizontal sample) would to a larger degree lead to these appearing as pearls on a string (see images of cells on Nanograss B).

### Image Processing

After the slice and view stack has been recorded several steps are required to convert it into a useful 3D dataset. To do this three steps are required: image scaling to correct for imaging on a slanted surface; alignment of the individual slices; and a coordinate transformation to match the original volume – all of which was done with the open source ImageJ software.

**Figure 1:**
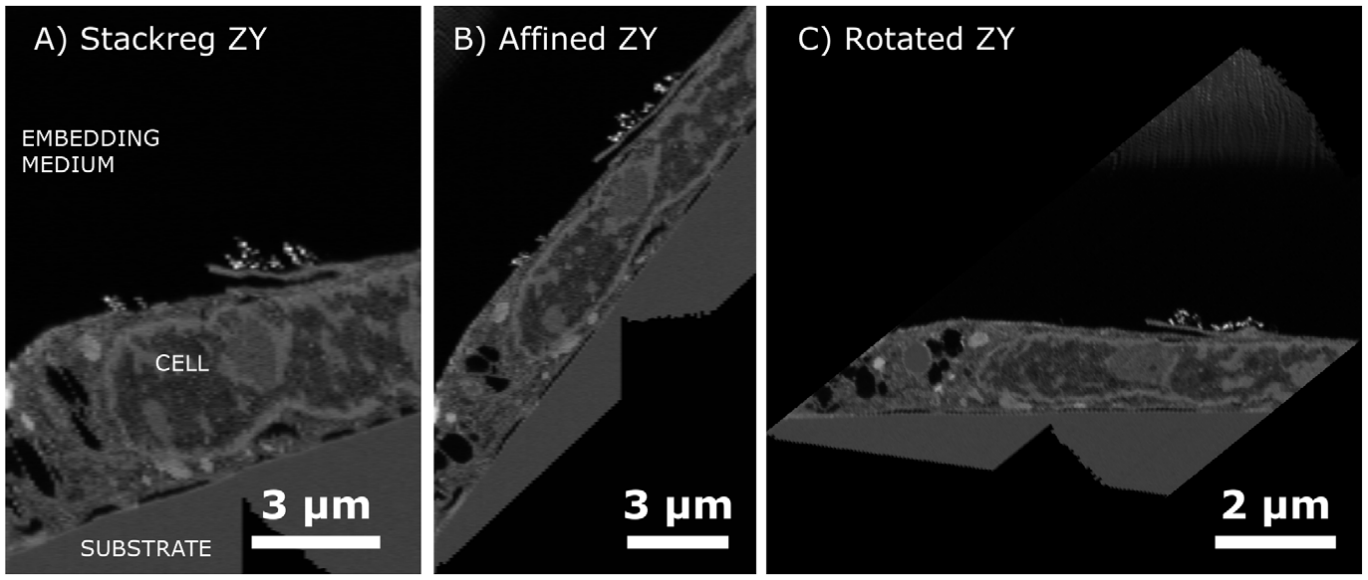
Side views of the non-tilted milling obtained image stack of a cell on glass showing the sequential processing operations’ effects. A) The individual slices have been aligned forming a fairly smooth image using stack-reg algorithm. B) Then the substrate is corrected such as to annul the effects of automatic E-beam shifts in the Slice and View program, resulting in a 52 degree substrate. C) Finally the image stack is rotated 52 degrees to represent the sample on the flat substrate having been cut at an angle.

The image is first scaled to obtain the image aspect ratio of the true slice surface instead of the compressed projection image from the tilted view. When image stacks are obtained, small random shifts between the slices occur, which is corrected with the stackreg plugin for imageJ. Lastly, affine volume transformations and rotation is performed to level the substrate to reshape the image volume to the original sample geometry. This procedure was done both for ordinary tilted milling, but also for non-tilted milling showing how a representative 3D stack can be obtained also when using non-tilted milling. To illustrate some of these transformations, the image stack obtained with non-tilted milling of a cell on glass can be observed from the side in Figure 1. For further detail refer to .

## Results

We first describe the blank sample with cells on glass and evaluate the FIB-EM quality. Next, the overview map of the observed interactions on nanostructures is presented, followed by discussions on interactions observed on different substrates.

### Cells on a Flat Substrate

For the NIH3T3 cells cultured on the unstructured blank sample of flat Pyrex glass and investigated with FIB-SEM the final stack’s resolution given by the pixels of the original image was 10 nm in X direction, 10 nm in Y direction and 100 nm in Z direction. Please note that the coordinates differ from that of typical cell microscopy as the FIB mills perpendicular to the sample making the X- and Y direction the width and the height of the cell respectively, instead of letting the Z direction denote the height of the cells as in confocal microscopy (cf. Text S2).

The correction procedure compromises the resolution in the Y direction as each pixel here have been multiplied with 1.27 as the SEM image is a projection of a 52 degree slanted surface (cf. Text S2), also see Text S2 for a comparison of as-imaged and corrected front view images.

**Figure 2:**
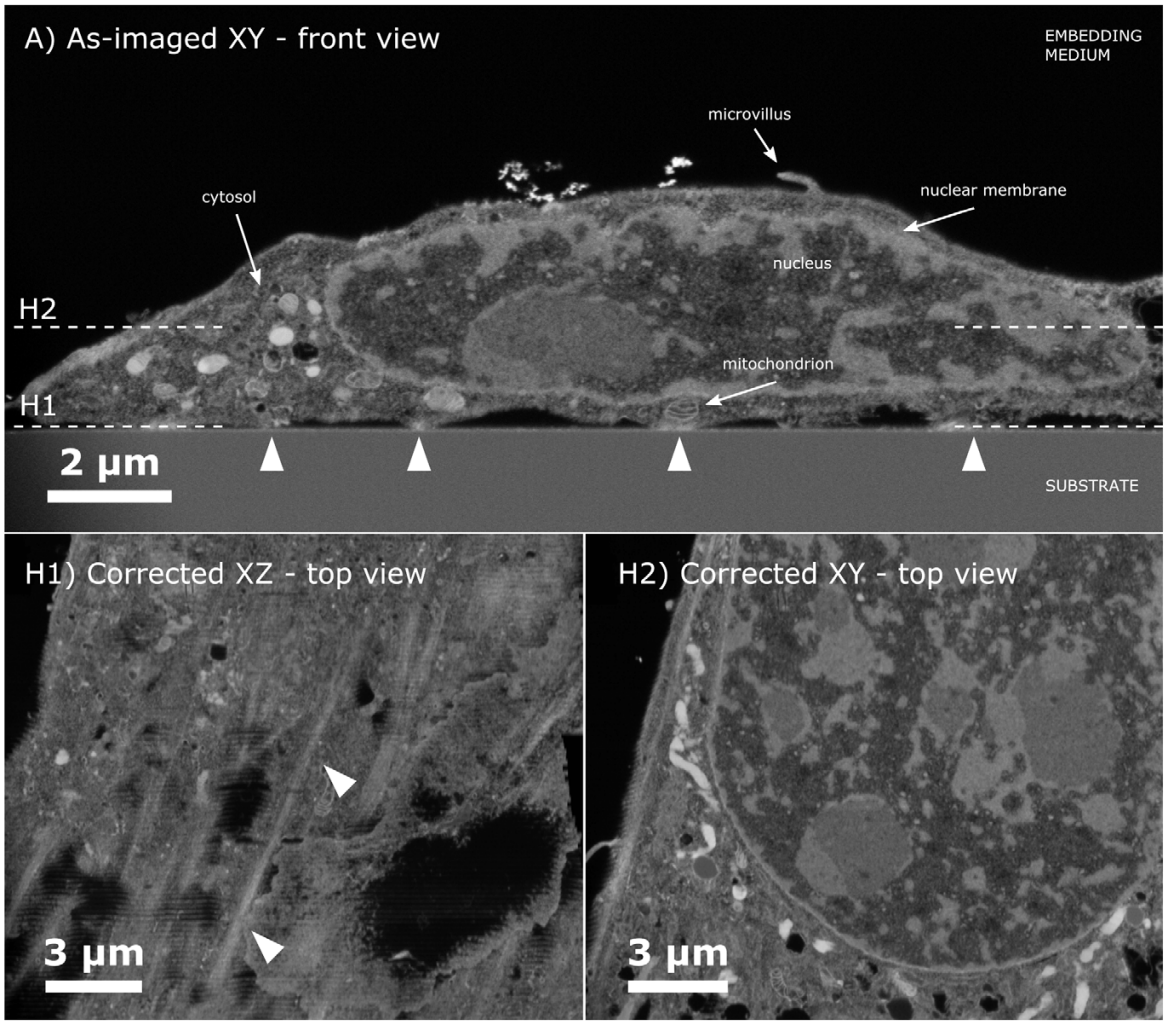
FIB-SEM image of cell on glass showing front view and top views. A) Front view shows a non-processed as-imaged slice of a cell on a glass substrate. One can see the nucleus, microvillius, and organelles such as mitochondria in the cell cytosol. The triangular arrows highlight the discrete points where the substrate and cell are in contact. Dashed white lines indicate the two height levels of the horizontal top view sections shown below. **H1)** Horizontal top view section of the cell close to the substrate level for the fully corrected stack, here it is seen that the cell contacts with the substrate in lines. The two white arrows show one such site where the cell touches the substrate. **H2)** A top view of the stack is shown higher up in the cell.

The image of the cell on glass (Figure 2) shows a cell with well defined organelles, membranes, and nucleus. Some vacuoles are seen in the cytoplasm of the cell on plain glass which is to be expected for fibroblasts, however no vacuoles were seen in the nucleus and vacuolisation as sign of apoptosis was not observed [44]. The reconstruction was done for 100 slices of non-tilted milling and only limited distortions are seen in Figure 2 H1 where horizontal ripples appear, whereas there are no distortions to mention in the section shown in Figure 2 H2. The ripples visible near the substrate are possibly due to imperfect alignment of the images using the stack-reg algorithm (cf. Text S2), which is less evident higher in the cell where there is no sharp transition between a flat substrate and the cell.

Even though no specific staining has been used to mark specific organelles or adhesion sites, the FIB-SEM method gives a high resolution three dimensional stack which here provide unique images. For instance when the stack has been corrected (and even before) it is possible to directly observe where the cell is in contact with the substrate. This can be observed both in the front view and top view, cf. Figure 2. From these images one can see that the cell interfaces with the substrate in lines, and not as points. This could be correlated with fluorescent labelled actin or focal adhesion stains to determine what these lines exactly represent [21], [45], [46]. Figure 2 A is a non-processed SEM image front view, since the processing steps diminish the resolution and image quality slightly (cf. Text S2); however, the same adhesion sites are observed in the fully processed stack as observed from Figure 2 H2.

### Cells on Silicon Nanowires, an Overview

**Figure 3:**
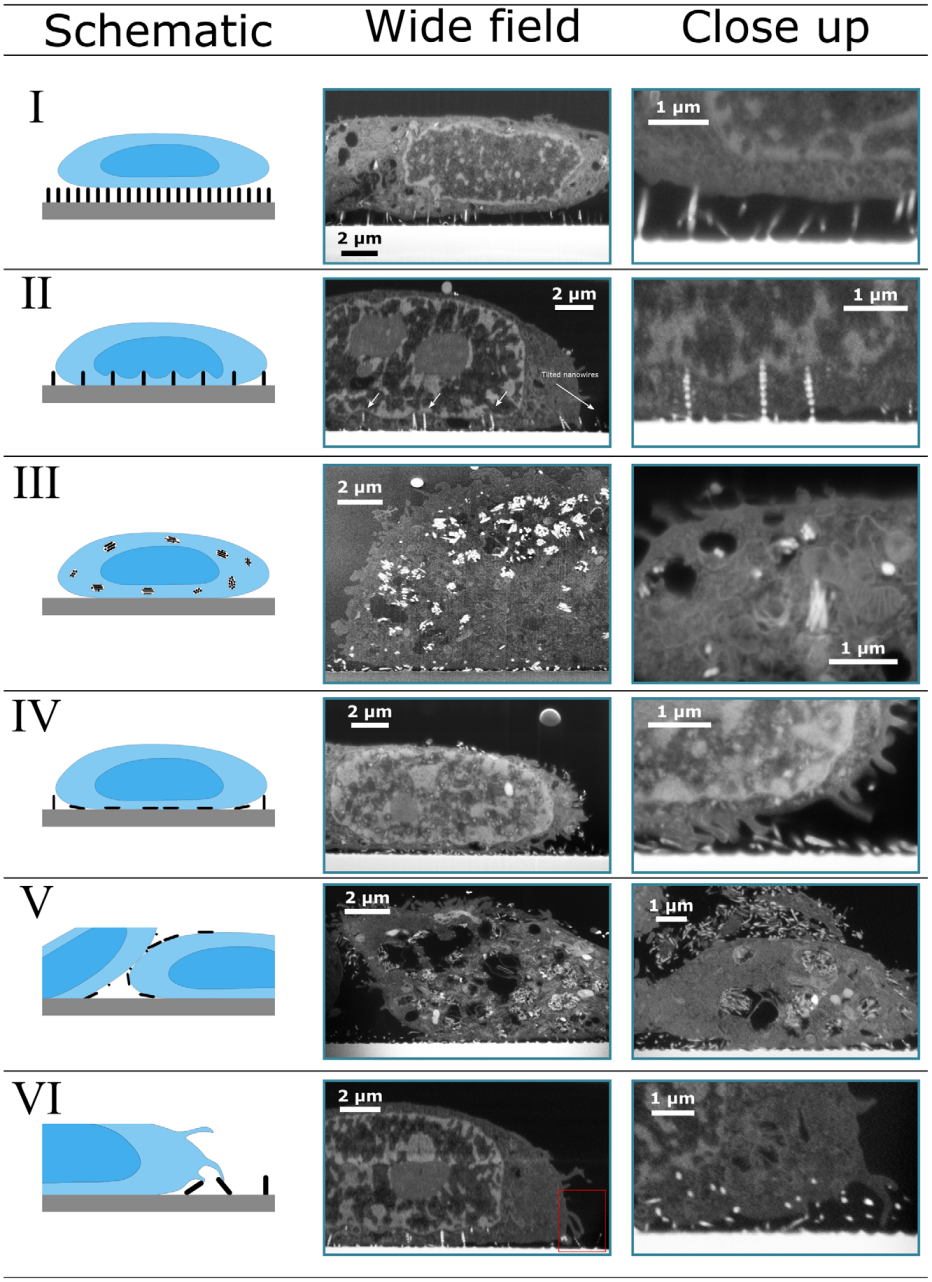
Map of the various cell-nanowire interactions observed. 6 cases are outlined with a schematic view and two supporting FIB-SEM images illustrating the case. Case VII, vacuolisation is to a large degree observed in images displaying Case III and Case VI.Inverted view can be found in Figure S3. The close-up images are either regions from the lower magnification image or higher resolution images from a different image.

During experimentation we have found several different ways that the cells interact with nanostructured substrates. In some instances the cell appeared to break off the nanostructures and engulf them, in other cases the nanowires appear to have penetrated the cell and in some the cells where observed lying on top of the nanostructures, these and more interactions are illustrated in Figure 3 (for those accustomed to TEM images, an inverted version can be found in Figure S3).

Based on the observations, we have defined 7 overall different interactions between the silicon nanowires and the cells, creating a starting point for a map of cell interaction with nanostructured substrates (Figure 3). It is a map showing cell morphology and not behaviour such as differentiation, toxicology or motility. The figure shows a schematic presentation of the interaction, a wide field image and a larger magnification of the same interaction (although not necessarily on the same cell or sample).

Many of the nanowire-cell interactions would not have been easily observable using light microscopy, ordinary SEM with CPD cells, or TEM of single microtomed slices. The images hence illustrate the unique capability of the FIB-SEM for imaging cells on nanostructured substrates. All these cases show that studying cells on nanostructures can lead to complicated interactions most likely affecting the cells in numerous ways compared to the blank glass sample.

The different morphological cases observed in the investigated cells:

**Case I** ***On top***: A nanowire forest working as a scaffold for the cell, where the cell has little to no contact with the underlying flat substrate beneath the wires, but rather the cell rests on top of the nanowires, which may create inwards bulging of the cell membrane. Observed for both types of nanograss, however more common in Nanograss B.

**Case II** ***Indented membrane***: The outer membrane may be indented to fold closely around the nanowire. The nanowire could penetrate the outer membrane although the present images do not clearly show if that is the case. In extreme cases, the nanowires were seen to indent the nuclear envelope. Some nanowires have been flattened, meaning that the pitch between the remaining nanowires have been increased, possibly allowing the cell to sink down on the remaining nanowires thereby allowing the nanowires to reach further into the cell than **Case I** and to affect the nucleus shape. This was only observed for Nanograss B.

**Case III** ***Uptaken:*** Nanowires, torn off from the substrate, and taken up by the cell. Wires can be found inside the cell in clusters within vesicles, an interaction seen in all cells, but more extreme in Nanograss A.

**Case IV** ***Flattening:*** Cell flattening weak nanowire forest. This effect is in particular seen in Nanograss A, but also seen to a lesser extent in Nanograss B.

**Case V** ***Interface:*** In some instances nanowires were torn of the substrate and would remain in the interface between cells. Only observed in Nanograss A.

**Case VI** ***Probing:*** All cells showed varying degree of microvilli or bleb like structures [47] probing the nanowires, but were in particular prevalent in Nanograss A.

**Case VII** ***Vacuolisation*** **:** Increased vacuolisation in the cell, in some cases these contained nanowires. Illustrated by images from **Case II** and **Case IV.**

These different cases are based on the FIB-SEM images from 10 different cells, 5 cells on Nanograss A and 5 on Nanograss B. Table 2 gives an overview of the interactions observed in the different cells. With the limited number of cells examined we cannot conclude much about the general frequency of these cases, nor give any indication as to the dynamic processes involved. For half of the studied cells, the cell was found to be on top of the nanowires (**Case I**), while **Case IV** was observed for the remaining 5 cells. All of the cells expressed multiple cases as can be seen in Table 2.

**Table 2:** Overview of the different cases observed in the 10 cells.

#	Substrate	Case I	Case II	Case III	Case IV	Case V	Case VI	Case VII
1	A			X	X		X	
2	A	X		X			X	
3	A			X	X	X	X	X
4	A			X	X		X	X
5	A			X	X		X	X
6	B	X		X			X	
7	B	X		X			X	
8	B	X		X			X	
9	B	X		X			X	
10	B		X	X	X		X	

Here it is evident that cells express more than one case and that some of these might be related, and in some instances be prerequisites for certain cases.

Only a single cell showed **Case II** behaviour, whereas uptaken nanowires (**Case III**) were observed in all the cases, however the most extreme cases were observed in the cells which also displayed a high degree of nanowire flattening (**Case IV**). In a single instance nanowires in between two cells were seen (**Case V**). As mentioned all 10 cells showed varying degree of nanostructure probing (**Case VI**), and 3 cells showed increased vacuolisation (**Case VIII**) while having rather extreme nanowire uptake (**Case III**).

### High Density Silicon Nanowires (Nanograss A)

**Figure 4:**
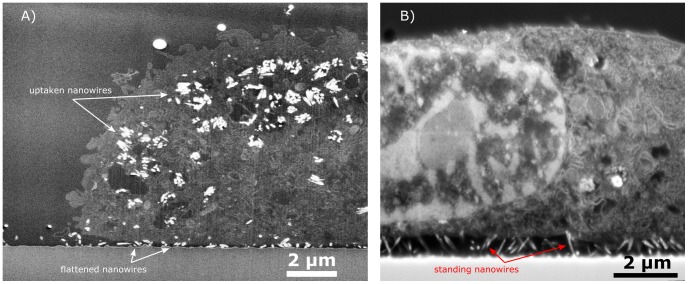
FIB-SEM image of cells on Nanograss A, illustrating different cell behaviours on the same substrate. A) FIB-SEM image showing a cell having engulfed broken-off nanowires, and clearly bent silicon nanowires underneath the cell. The nanowires are closely packed in tightly formed clusters inside what appears to be vesicles. **B)** Another cell on the same substrate, this time the nanowires have been bent by the cell but not completely flattened.

In the case of cells cultured on Nanograss A, the images indicate that the nanowires did not have sufficient mechanical strength to withstand forces exerted by the cell. The cells would typically flatten the nanowires (**Case** **IV**) and engulf them (**Case III**), as seen in Figure 4 A. Nanowires were also observed stuck in between two adjacent cells’ membranes (**Case** **V**). Five cells were imaged (not whole cell 3D slice and view), all of them showed varying degrees of nanowire uptake into organelles appearing like vesicles (Figure 4). Two cells showed significantly lower concentration of engulfed NWs than Figure 4 A. Four cells almost completely flattened the nanowires, whereas the remaining was situated on top of the nanowires. Generally the substrate also induced a high level of microvilli activity probing the nanowires as illustrated by **Case VI**, and in some instances increased vacuolisation as **Case VII**.

Once inside the cell, nanowires tended to agglomerate in vesicles or areas with a distinct lack of heavy metal staining. It is not clear whether the agglomeration of the nanowires was caused during endocytosis, where the cell uptakes the nanowires in vesicles to avoid direct contact with the cytoplasm, or the nanowires had agglomerated prior to intake due to nanowire clustering or a wetting effect.

In some instances the nanowires appear as hollow cylindrical objects with ellipsoidal cross section when cut at oblique angles; while direct end-on imaging provides round cross sections (cf. Figure 4 and Figure S4). However, the nanowires are not expected to be hollow, as they are created by a top-down processing approach by etching of a monocrystalline silicon substrate. This observation can be explained with the formation of a native silicon dioxide at the nanowires’ surfaces or even some plausible oxide growth during processing. Silicon dioxide has a higher secondary electron yield than bare silicon, in fact, K. Okamoto in 1980 showed how measuring the ratio in secondary electron signals from bare silicon and silicon oxide could be used to determine the thickness of the oxide [48]. This effect means that surface oxide would yield a larger generation of secondary electrons than bare silicon, resulting in higher brightness. The images showing ‘hollow’ nanowires have been obtained with an in-lens system from FEI, which also captures secondary electrons. This explains why the nanowires have a bright oxide ring around an inner silicon core, producing the hollow looking nanowires. This is not observed in the other images presented in this paper as a designated backscatter electron detector has been used (for instance see Figure 3, **Case III** close up), limiting the visual effect of the increased secondary electron generation from the oxide.

Nanowires could to some degree have been flattened during handling or perhaps be a result of cell deformation or shear forces during the embedding process. However as the nanowires in Figure 4 B show, the nanowires appear to be tilted in either direction indicating that it is due to a specific cell interaction with microvilli instead of overall cell volume changes or any dislocation during the embedding procedure. Furthermore, the nanowires outside the range of the cells are freely standing up (Figure S5), and images of embedded nanowires having endured the same treatment can be seen in Figure S1

### Low Density Silicon Nanowires (Nanograss B)

For the cells cultured on Nanograss B many of the same phenomena were observed as with Nanograss A. For the 5 investigated cells, 4 of them were found to be lying on top of the silicon nanowires as illustrated by **Case I** (see Figure 3 or Figure S6). To some extent nanowires were also bent underneath the cell as **Case IV**. Like Nanograss A, nanowires were found inside the cells as described by **Case III**, and microvilli interaction with the nanograss was observed (**Case VI**), albeit both cases appear to be less prominent compared to Nanograss A. Unique to a single investigated cell, nanowires were seen indenting the nuclear membrane (**Case II**).

**Figure 5:**
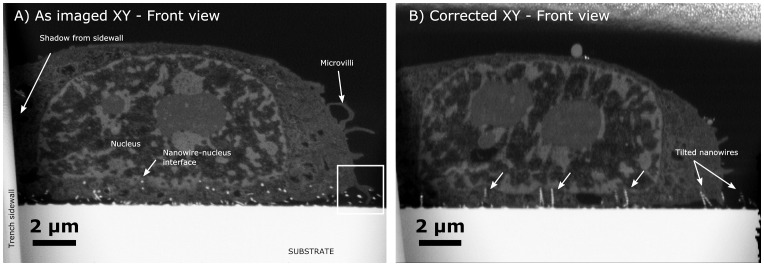
FIB-SEM images of a cell on Nanograss B. A) As imaged (y-corrected) slice showing the nanowires which appear as white dots due to insufficient sampling frequency. Also worth noting is the example of **Case III** behaviour with microvili probing the nanograss as outline by the white frame. **B)** The fully corrected stack can be seen, here the stack has been fully corrected such that independent white dots representing a single nanowire align, illustrating the suboptimal sampling frequency. In some cases the nanowires are not shown from top to bottom as they are slightly tilted compared to the section, quite possibly due to interaction with the cell. Also seen is how the nucleus is avoiding the nanowires (white arrows), and the rippling artefacts which occurs in the corrected front view as previously mentioned.

For one cell cultured on low density silicon nanowires, the nanowires appear to enter the cytosol and penetrate the cellular membrane (cf. Figure 5). However, the resolution of the images is not sufficient to unambiguously determine whether the nanowires are enveloped by a membrane or not. The nuclear envelope appears not to have been penetrated but rather indented and remains on top of the nanowires (cf. Figure 3) much like the case for the outer membrane when cells lie on top of nanowires (**Case I**). Also observed on the figure is the difference between an ‘as imaged’ and corrected image. On the ‘as imaged’ slice, the nanowires appear as isolated white dots due to the nanowires being cut by the FIB at a non-normal angle. In the fully corrected stack, the nanowires appear as a string of white dots, which illustrates a case of insufficient Z-resolution (excessive slice thickness) in slanted milling. The slices were made at an interval of 100 nm for this particular sample, exceeding the diameter of the nanowires (approximately 70 nm). This means that the nanowires cannot be fully represented in the recreated volume and accordingly takes shape as a string of spheres. Figure 5 therefore illustrates the suboptimal sampling frequency which gives rise to artefacts in the reconstruction, even though important cellular features are still discernible.

**Figure 6:**
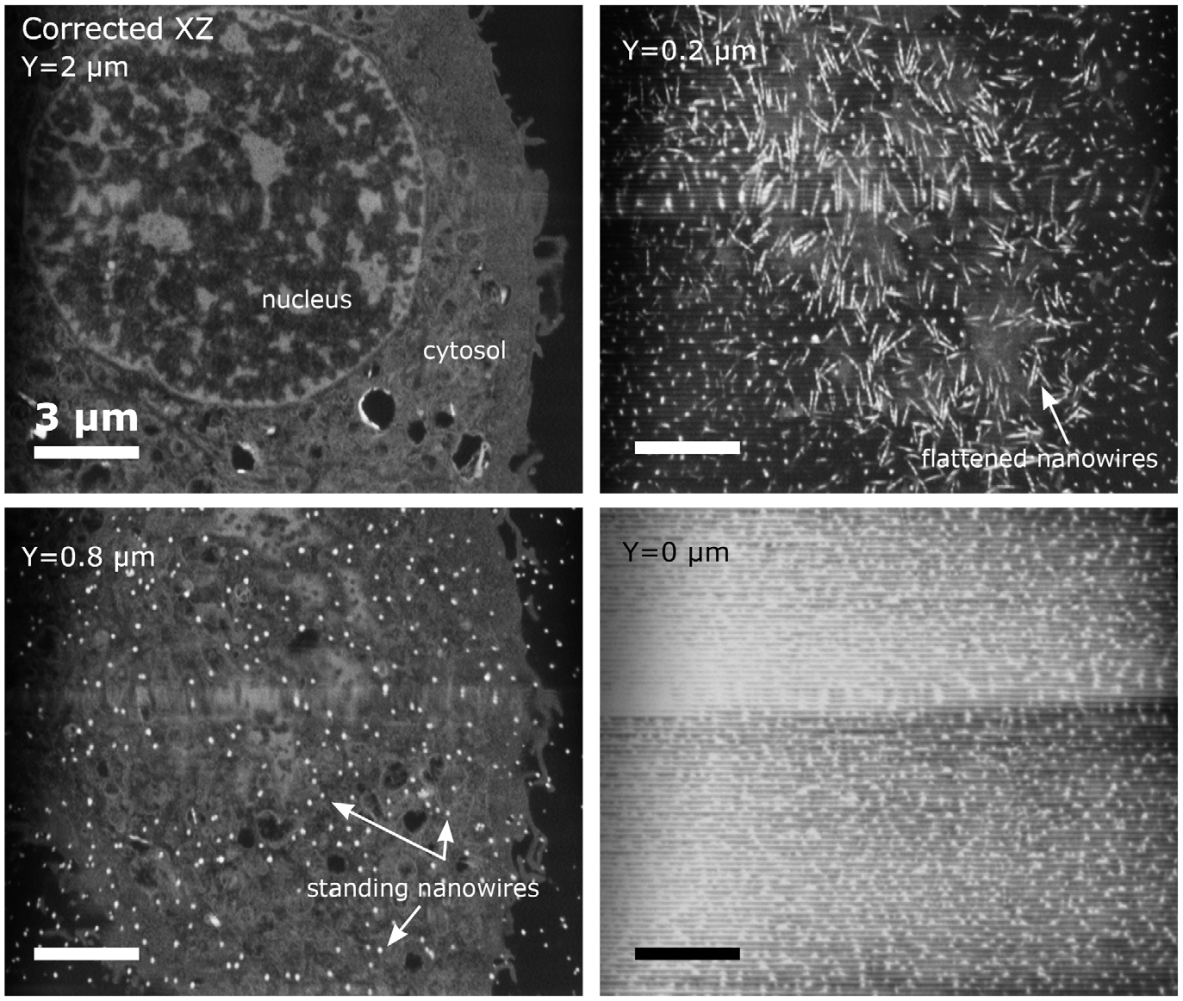
Image series showing the top view FIB-SEM image of the same cell as in Figure 5 on Nanograss B. The sections have been made from 5 µm above the substrate to 0.25 µm above the substrate. This illustrates the major forces in play, clearly showing how several nanowires where bent underneath the cell leaving only a few left to indent the nucleus membrane.

The reason why the nanowires in the corrected image in some cases does not show the entire length of the nanowire (top-to-bottom) is that the nanowires were tilted compared to the imaging plane (cf. Figure 5). In general, the cell appears to have exerted significant force to the nanowires, in some instances slightly tilting them, but in other bending them such that they lie under the cell (cf. Figure 6).

This stack is a good example of multiple behaviours observed in a single cell with **Case II**, **Case IV** and **Case VI** behaviour. The cell’s nuclear membrane is indented by the nanowires (**Case II**), but it also flattens some of the nanowires (**Case IV**) while probing the nanowires (**Case VI**). Flattening of nanowires is best seen in Figure 6 where horizontal top view sections are displayed, again illustrating the unique volume viewing quality of the FIB-SEM.

Compared to Nanograss B, the dense silicon nanowires in Nanograss A seem more fragile even though their characteristics are fairly similar except for their density; Nanograss A was to a larger extent not able to withstand adhesion forces exerted by the cell. In addition, Nanograss A also seemed to have higher silicon uptake and it is accordingly fair to assume that it would have a significant influence on the cells, and possibly also induce apoptosis as the vacuolisation in some cells suggest [44]. It is unclear whether the nanowires are uptaken only directly from the substrate’s surface or whether they are taken up from the surrounding solution, which could be possible for **Case V** where loose nanowires are situated in the interface between cells.

## Discussion

The cells breaking up and bending the nanowires implies a certain amount of force applied to the substrate. Regarding the forces in play responsible for the nanowire perturbations seen in the different cases, it is a well-known issue that capillary forces can result in nanowire clustering, which is why samples were kept wet after the first wetting [43]. It is evident from the images showing standing nanowires underneath cells and from the blank nanowire samples (Figure S1) that capillary forces and processing did not induce extensive clustering or breaking of wires, although some collapsed nanowires are always to be expected during fabrication and processing.

Several studies have been made on forces involved in nanowire bending and breaking. Using AFM measurements on silicon nanowires Hoffmann *et al*., measured standing silicon nanowires bending strengths. For a variety of nanowire sizes (diameters from 90–190 nm) with height width ratios between 4 and 12 they obtained a maximum force before fracture between 1 µN and 7 µN [49]. The relationship between the maximum force and the aspect ratio of the nanowire appear to be linear, assuming similar conditions for our silicon nanowires, the fracture force for the used nanowires can be calculated to be around 300 nN as a rough estimate.

If we only look at gravitational forces, a cell used in this experiment is estimated to be no heavier than 5 ng (mass of a HeLa cells is about 2–3 ng). This will give rise to a force of about 50 pN when ignoring the buoyancy. Assuming that the density of the cell is 10% higher than the medium, this only results in a gravitational force of 5 pN, which according to the rough estimate of the nanowire strength should not be sufficient to flatten or break the nanowires as we have observed and would in the observed cases also be distributed over many wires. The cells can however apply considerable in-plane forces, single focal adhesion site (FAS) forces of 10–30 nN have been reported by Balaban *et al*., and in addition a single cell has been shown to be able to resist a transverse pulling force of 450 nN without detaching from the substrate [45]. Forces measured on single pillars have been reported in the 50 nN range for fibroblasts [50], [51]. Measuring the lateral deflection of silicon nanowires for CPD dried cell on the substrate, Li *et al*., reported cell traction forces in the µN range for three different cells lines [52]. Munevar *et al*. reported average traction forces for migrating fibroblast in the order of 1–5 µN per cell, by measuring the displacement of substrate integrated beads in the wet state [53].

We speculate that these numbers from the literature describing cell forces could indicate that focal adhesion sites and cell movement do have the necessary strength to cause some of the effects which we have observed. In most cases (6 out of 10 observed), the cell was seen lying on top of the nanostructures where cell forces were not sufficient to bend a large amount of nanowires. Migrating cells, however, might incur higher traction forces and possibly be able to flatten larger areas of nanowires as seen in some instances in literature [53].

Regarding the presented map it should seen as a first attempt at organising the cell-nanostructure interactions as has been done e.g. with endocytosis of nanoparticles [11], [12]. Many of the observed cases we have observed have also been seen or hinted in literature on a wide variety of cell types and substrates. For instance cells have been found to reside on top of nanostructures (**Case I**) in several papers [14], [22], [28]. Hanson *et al*. used ultramicrotomed thin sections to describe the interface between cortical neurons on nanopillars. By varying the dimensions and density of the pillars they found cells that lied on top of the structures (**Case I**) and also how a cell could sink down onto the pillars resembling **Case II** [22] though they did not observe nuclear indentation. TEM images of nuclear indentation (**Case II**) were obtained by Hai *et al*., where spine shaped gold protrusions indents the nucleus membrane [8].

Regarding the uptake of 1D nanostructures (**Case III**), a lot of the focus has been on carbon nanotubes and their possible toxicological effects [54], [55], but other materials has also been investigated [56]–[58]. Common for these studies is that the nanostructures were in some form of suspension, while uptake of initially substrate fixed nanowires does not appear to have been reported elsewhere.

As discussed above, certain cell types are able to exert significant forces on nanostructures [45], [50], [51]. But the structures they used were quite robust as they were used for force measurements so the same flattening effect (**Case IV**) was not seen to such an extreme degree. Cell probing of the nanostructures (**Case VI**) have been seen in multiple instances [27], [59], whereas increased vacuolisation (**Case VII**) due to nanowire uptake to our knowledge has not been reported, but increased vacuolisation due to other perturbations have been documented [60], [61]. By organising the interactions one might find correlations between the complex interactions and better our biological understanding of the underlying pathways as has been done with endocytosis of nanoparticles [11], [12].

### Conclusions

FIB-SEM imaging of cells on nanowires provides a unique 3D imaging modality, and has the ability to resolve a variety of different internal and external interactions between cells and a nanostructured substrate, based on embedded and heavy metal stained samples. The method presented show interactions with a resolution not obtainable with confocal/fluorescence microscopy, and allows 3D reconstruction of the sample not easily obtained with TEM.

Regarding the trueness of our images, many of the interactions were seen in multiple cells. In addition, the ultrastructure of the cells seems well preserved with visible cell membranes, nuclei and organelles. The fact that the nanowires did not collapse during sample handling also indicates that the images provide a fair representation of what could actually have taken place *in vitro*.

It was also shown that non-tilted FIB-SEM milling could be performed and the stack be reconstructed with the developed method using the freely available software (ImageJ). For non-tilted milling, one should be mindful of having sufficient sampling frequency, while tilted milling was less susceptible to the issue when imaging vertical nanostructures.

Even though the two nanowire substrates were quite similar, differences in cell behaviour could be observed. Nanograss A appeared to have more fragile nanowires which more easily broke of the substrate, were engulfed by cells or simply flattened underneath the cell. Nanograss B in contrast proved to be a more sturdy substrate, but still nanowires were flattened, tilted, and uptaken. The difference between these two substrates seems to be linked to the density of nanowires, where Nanograss A had a higher density of wires leading to groups of nanowires sticking together and more nanowires being bent. In either case both substrates has a strong perturbing effect on the cell morphology.

As we have shown, the vast phenotypic variability gives a large difference in cell appearance on nanostructures, and illustrates that single cell investigation is not sufficient. Quantification of cell-nanostructure interactions thus requires careful statistics by methods with higher throughput, for instance light microscopy methods, and then supported with representative imaging with FIB-SEM, which is outside the scope of this paper. This study provides an overview map that serves as a starting point for development of high throughput light microscopy methods capable of investigating cell-nanostructure interactions taking due care of the many possible types of interactions. Additionally, to make the map more complete we suggest using TEM for higher resolution imaging cell-nanostructure interfaces imaging as it can be used to resolve how the cell membrane bends and if it has been penetrated, thus expanding on the previous work of [8], [22].

Investigations using electron microscopy have lead to an increased understanding of the vast complexity of cellular membrane anatomy; this is particular true for the different nanoparticle uptake pathways in cells which have been observed [11]. The field of endocytic pathways has evolved from a singular focus on clathrin-mediated endocytosis to 10 different mechanisms [11], illustrating the complexity of cellular membrane transport. Likewise the case of uptaken nanowires will likely have numerous pathways, and the way the cells interact with anchored nanostructures may cause novel pathways to come into action. Furthermore, the 7 cases presented in the map should by no means be interpreted as an exhaustive list, the vast complexity of endocytic pathways illustrates that much research is warranted into this field.

Our work focused on ultrastructural FIB-SEM investigations of cell-nanostructure interactions. To attain a greater understanding of the interactions we would suggest extensive correlated studies with fluorescent markers, and the usage of molecular techniques to block certain molecular mechanisms to be able to pin-point the biological processes involved, using the presented map as a starting point.

## Supporting Information

Figure S1 SEM images of the two types of nanograss substrates used. The two upper images show ordinary SEM images of the substrates, whereas the two below show the nanograss substrates having endured the embedding process. The embedded substrate images show standing nanowires and some which have tilted like the non embedded ones.
(TIF)

Figure S2 Examples of SEM images taken from above at 30 kV, showing cells lying on a nanostructured substrate underneath an embedding layer. Left, image of cells (lighter grey) that can be found from atop on a good sample obtained with backscatter detector. The small white dots are small defects in the surface of the embedding layer. Right, secondary electron signal also shows visible cells underneath the epon, but with less contrast.
(TIF)

Figure S3 Overview image where the EM images have been inverted.
(TIF)

Figure S4 Illustrating the hollow circular and cylindrical cross sections observed depending on the angle of milling and the orientation of the nanowire. Notice how the nanowires appear to be hollow, whereas they are expected to be solid.
(TIF)

Figure S5 Nanograss A showing standing nanowires next to the cell.
(TIF)

Figure S6 Images illustrating the variance also observable for Nanograss B. To the left internalised nanowires are shown, and to the right a cell resting on top of nanowires can be viewed.
(TIF)

Text S1 Supplementary information describing the embedding protocol used for embedding cells on substrates.
(DOCX)

Text S2 Here the image processing after the slice and view process is explained. The developed steps for data processing of an image stack obtained both on a tilted and non-tilted substrate is described.
(DOCX)
