# Supplementary figures and images for: Mapping the Complex Morphology of Cell Interactions with Nanowire Substrates Using FIB-SEM
Source: PLoS One. 2013 Jan 9;8(1):e53307. doi: 10.1371/journal.pone.0053307 (PMC3541134; doi:10.1371/journal.pone.0053307)

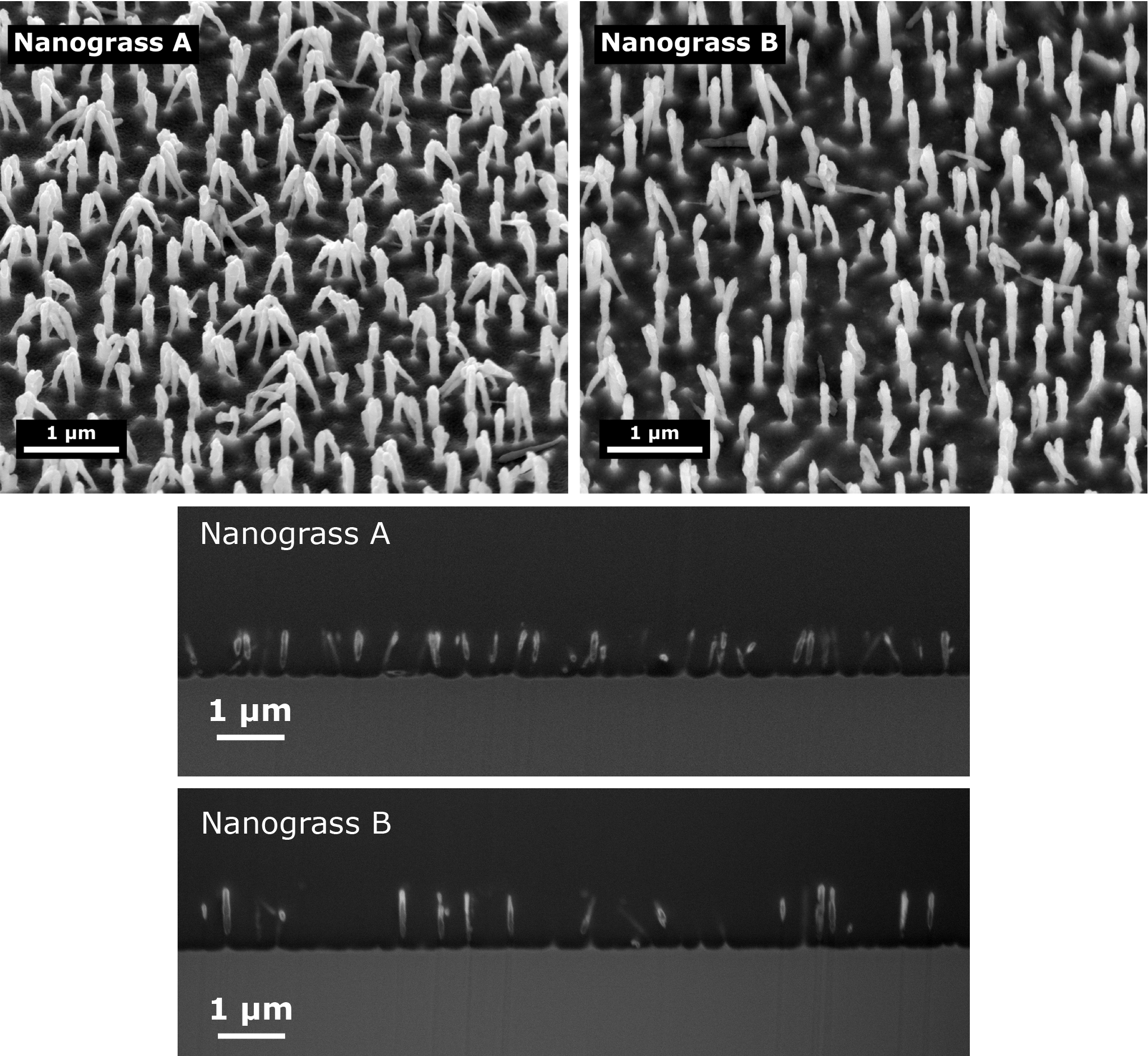

Supplement: Figure S1 — SEM images of the two types of nanograss substrates used. The two upper images show ordinary SEM images of the substrates, whereas the two below show the nanograss substrates having endured the embedding process. The embedded substrate images show standing nanowires and some which have tilted like the non embedded ones. (TIF) [file pone.0053307.s001.tif]

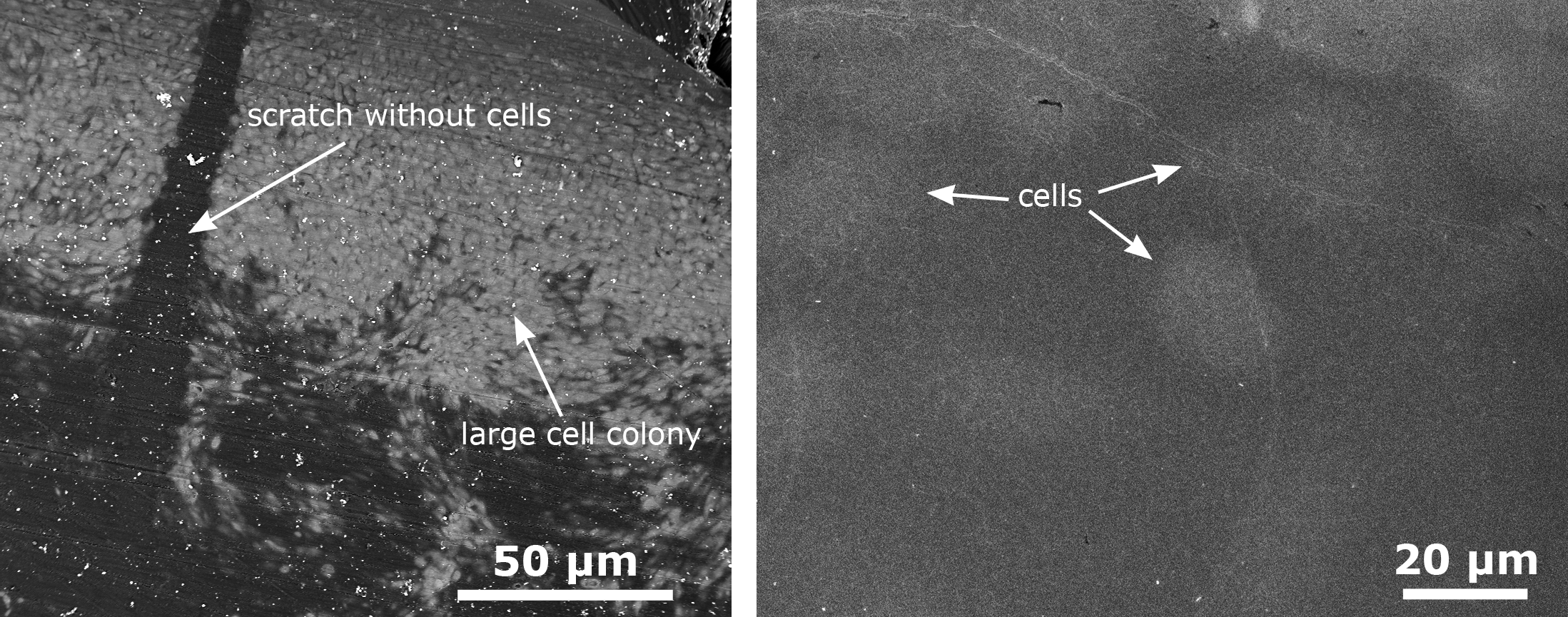

Supplement: Figure S2 — Examples of SEM images taken from above at 30 kV, showing cells lying on a nanostructured substrate underneath an embedding layer. Left, image of cells (lighter grey) that can be found from atop on a good sample obtained with backscatter detector. The small white dots are small defects in the surface of the embedding layer. Right, secondary electron signal also shows visible cells underneath the epon, but with less contrast. (TIF) [file pone.0053307.s002.tif]

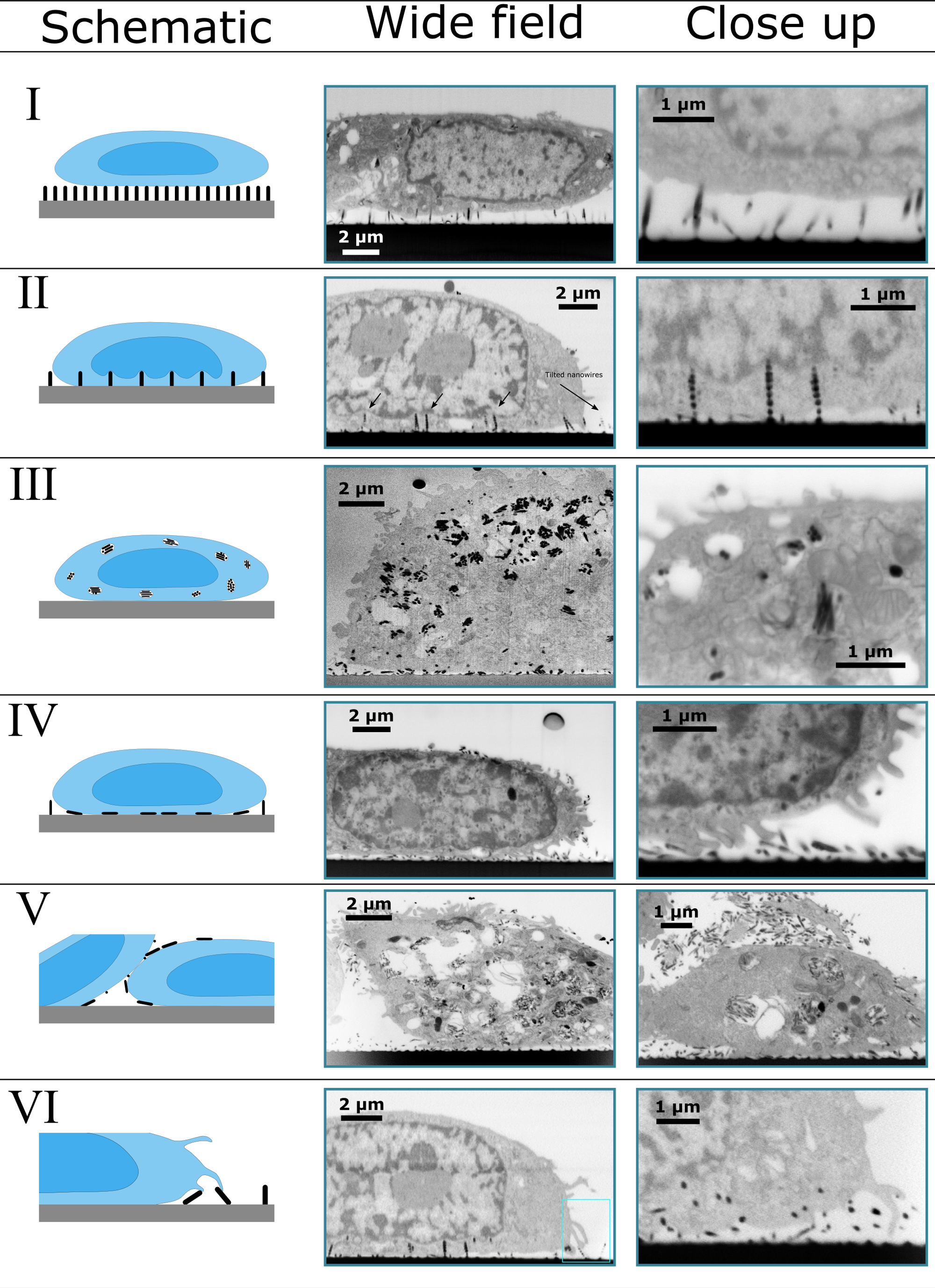

Supplement: Figure S3 — Overview image where the EM images have been inverted. (TIF) [file pone.0053307.s003.tif]

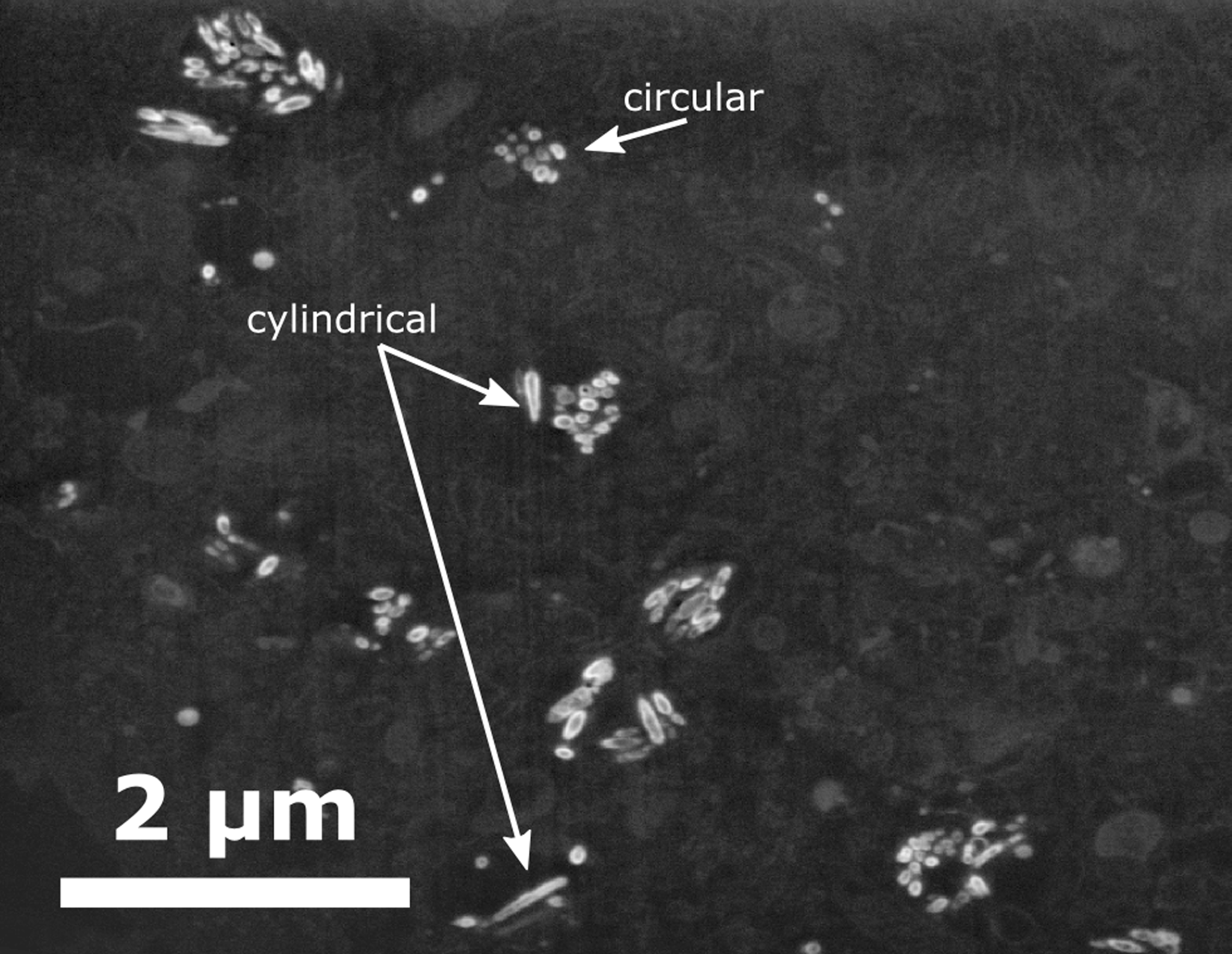

Supplement: Figure S4 — Illustrating the hollow circular and cylindrical cross sections observed depending on the angle of milling and the orientation of the nanowire. Notice how the nanowires appear to be hollow, whereas they are expected to be solid. (TIF) [file pone.0053307.s004.tif]

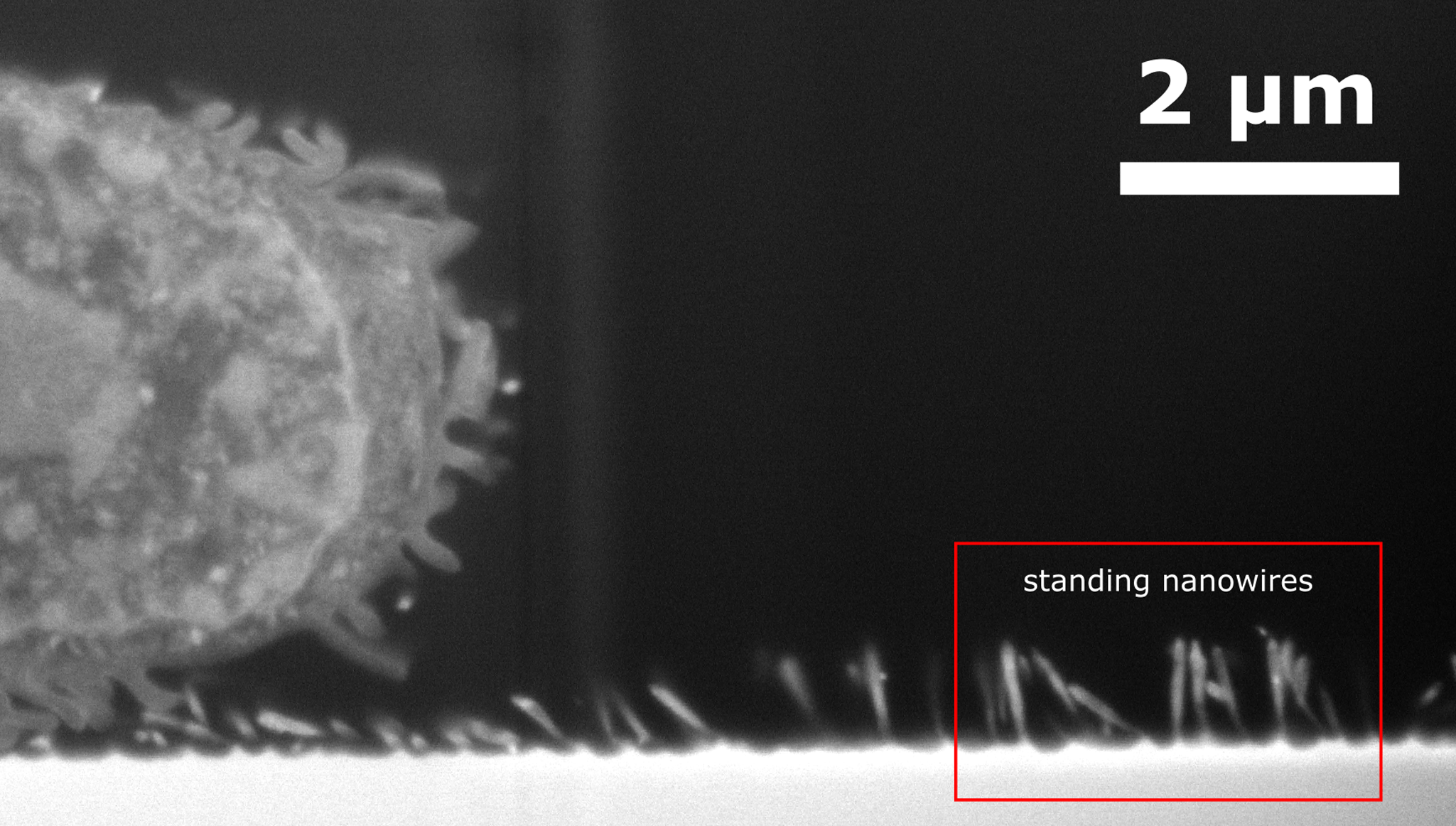

Supplement: Figure S5 — Nanograss A showing standing nanowires next to the cell. (TIF) [file pone.0053307.s005.tif]

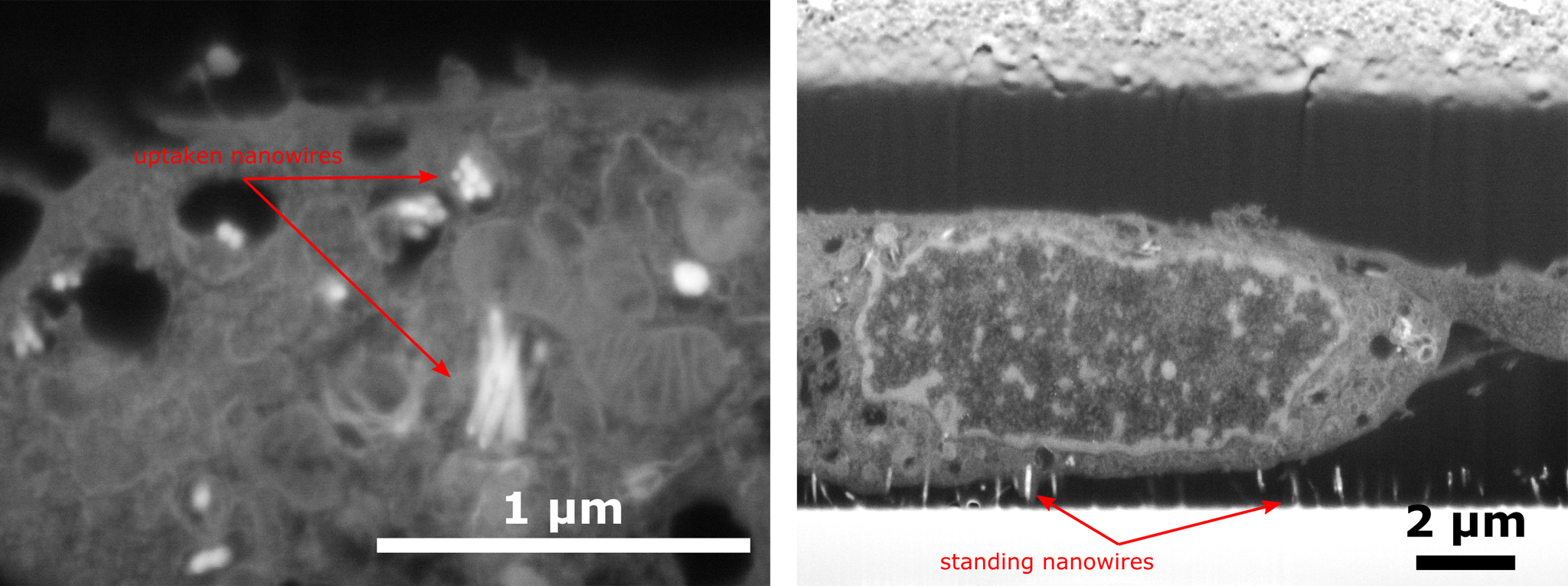

Supplement: Figure S6 — Images illustrating the variance also observable for Nanograss B. To the left internalised nanowires are shown, and to the right a cell resting on top of nanowires can be viewed. (TIF) [file pone.0053307.s006.tif]
