# Supplementary material for: Mapping the Complex Morphology of Cell Interactions with Nanowire Substrates Using FIB-SEM
Source: PLoS One. 2013 Jan 9;8(1):e53307. doi: 10.1371/journal.pone.0053307 (PMC3541134; doi:10.1371/journal.pone.0053307)
Supplement: Text S1 — Supplementary information describing the embedding protocol used for embedding cells on substrates. (DOCX) [file pone.0053307.s007.docx]

#### Text S1 - Protocol

Fixation 2% GDA (0.2 M) + 0.05 M cacodylate buffer (total 300 mOsm) Min. 1 hour

Flush 0.15 M cacodylate buffer 2 X 30 min

Post-fix/stain 1 % OsO4 + 0.12 M cacodylate buffer 1 hour

Rinse Milli-Q 2 X 10 min

Mordant 1%wt tannic acid in Milli-Q 1 hour

Rinse Milli-Q 2 X 10 min

Stain 1%wt Uranyl acetate in Milli-Q (can be left overnight) 2 hours

Dehydration 70% ethanol (can be stored in 70%) 2 X 10 min

Dehydration 96% ethanol 2 X 10 min

Dehydration 100% ethanol 2 X 10 min

Dehydration Propylene oxide 2 X 10 min

Embedding 1:3 Epon / Propylene oxide 30 min

Embedding 1:1 Epon / Propylene oxide overnight

Embedding 3:1 Epon / Propylene oxide 1 hour

Embedding 100% Epon 2 hours

Curing Cure at 60 deg. 48 hours


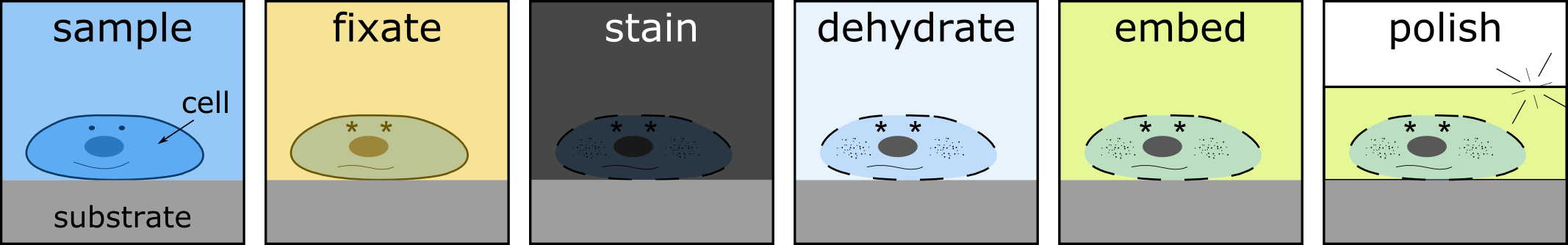


Figure 1 illustrating the embedding procedure from fixation to embedding.
