# Supplementary material for: Mapping the Complex Morphology of Cell Interactions with Nanowire Substrates Using FIB-SEM
Source: PLoS One. 2013 Jan 9;8(1):e53307. doi: 10.1371/journal.pone.0053307 (PMC3541134; doi:10.1371/journal.pone.0053307)
Supplement: Text S2 — Here the image processing after the slice and view process is explained. The developed steps for data processing of an image stack obtained both on a tilted and non-tilted substrate is described. (DOCX) [file pone.0053307.s008.docx]

#### Text S2 – image processing

After the slice and view stack has been recorded several steps are required to convert it into a useful 3D model. To do this **three** steps are required: scaling of the Y-direction, alignment of the individual slices, and a coordinate transformation to match the original volume – all of which has been done with the open source ImageJ software^[[1]](#footnote-1)^.


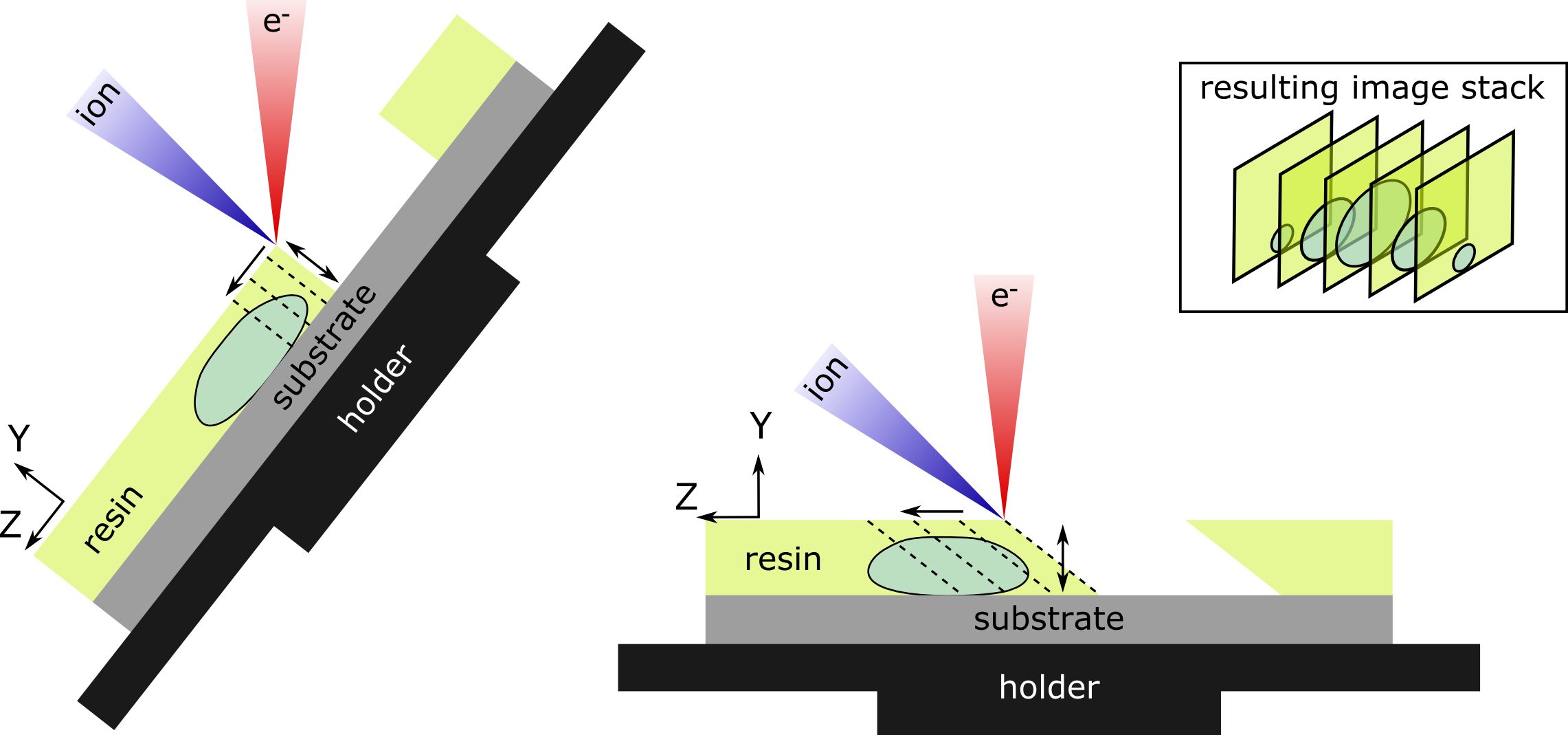


Figure 2 Left, the usual method in FIB-SEM is illustrated, here the sample is tilted towards the ion beam, resulting in normal incidence milling. Right, instead of tilting the sample, the ion beam is milling at an angle. The insert shows how an image stack is build up, representing the sample. Notice the Z-direction is parallel to the substrate.

First, as the FIB is at an angle to the SEM, each slice is tilted versus the e-beam; therefore the obtained image for each slice is a compressed projection, which needs to be stretched to match the actual aspect ratio of the original slice. With an angle of 52 degrees between the FIB and SEM, this correction in the Y-scale amounts to approximately 1.27 – simply performed in ImageJ.

Second, the SEM imaging will have small random shifts between the slices, most likely due to surface charging. That creates a significant distortion of the 3D geometry when the slices are stacked together. A pyramid registration alignment algorithm [1], the stackreg plugin^[[2]](#footnote-2)^ was used for matching of the consecutive slices.

Third, depending on the amount of automatic beam shift between slices the stack will in the ZY plane exhibit an angled substrate. For the tilted sample the data is skewed using the affine function to level the substrate. If the automatic beam shift had matched the displacement of the substrate in the Y-direction exactly as it was milled this had not been necessary. For the non-tilted sample however skewing of the stack to level the substrate would lead to a distorted stack. Instead the stack is skewed to obtain a substrate angle of 52 degrees after which is rotated to represent the original volume. Refer to (Figure 3 and Figure 5) to see the different transformations - these transformations are performed using the affine function in ImageJ [2]^[[3]](#footnote-3)^.


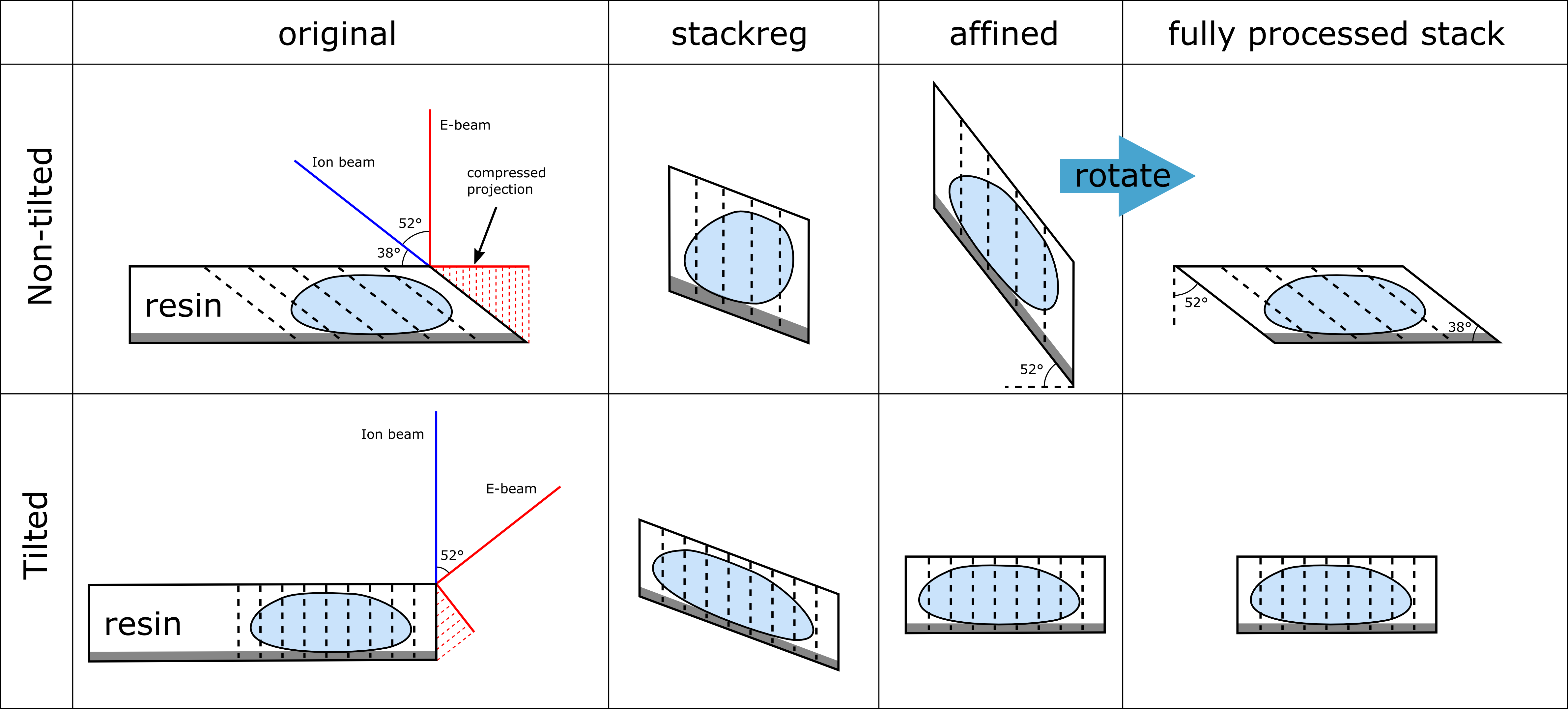


Figure 3 The different transformations made to obtained image stack to make it represent the original volume.

To illustrate how the transformations look for a non-tilted sample, the individual steps for the non-tilted cell on a glass substrate is shown in Figure 5 and Figure 6.

An image stack was obtained with a non tilted sample, a single slice and the stack seen from the side can be seen in Figure 4. This stack has to be post processed as outlined above, that is, it has to be: stretched in the y-direction, individual slice aligned using the stackreg algorithm, the substrate corrected using the affine function, and then the stack has to be adjusted for not being obtained with normal angle milling (cf. for the side view showing the different effects of the post processing).


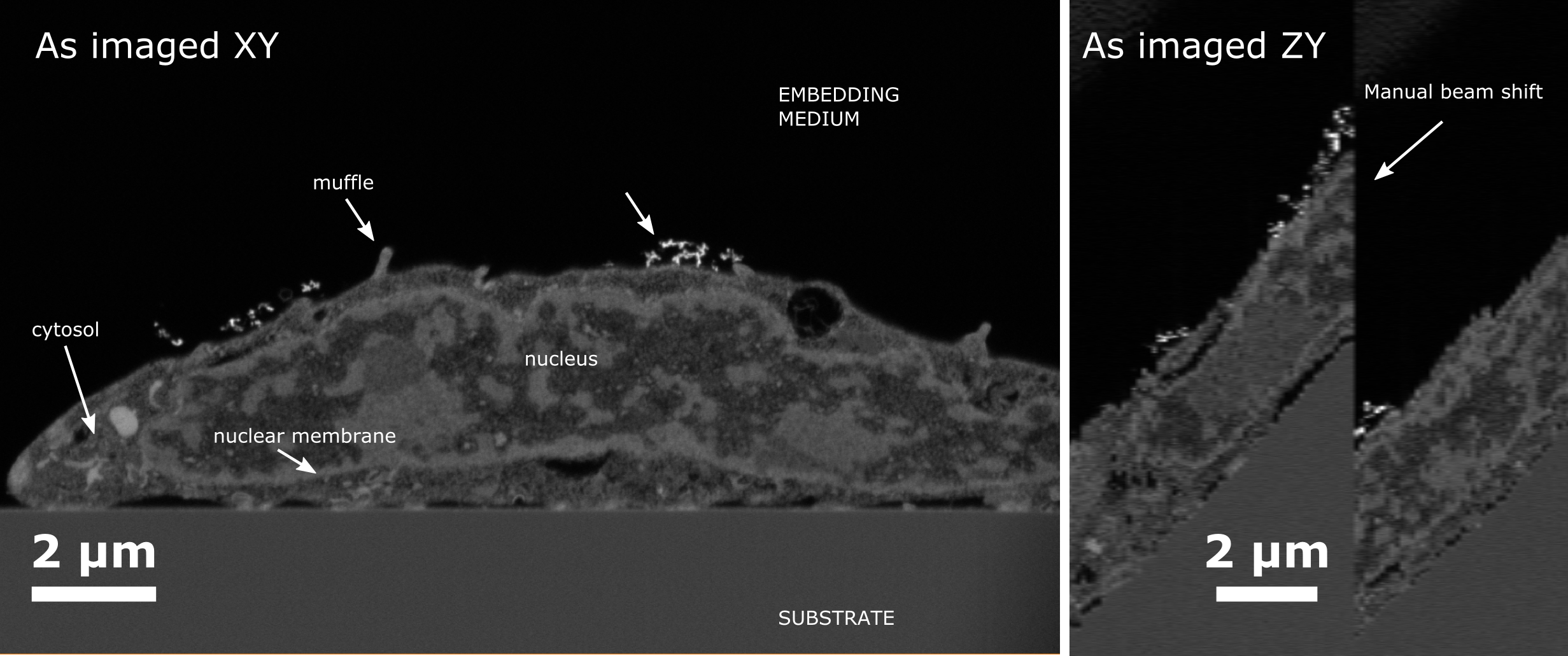


Figure 4 Left, a single slice of the raw data can be seen (as imaged), showing how the cell is situated on the substrate and some internal structures in the cell can be seen. Right, here the raw image stack is seen from the side, no post processing done. A large manual beam shift was performed during imaging resulting in the large jump, together with smaller slice to slice variations.


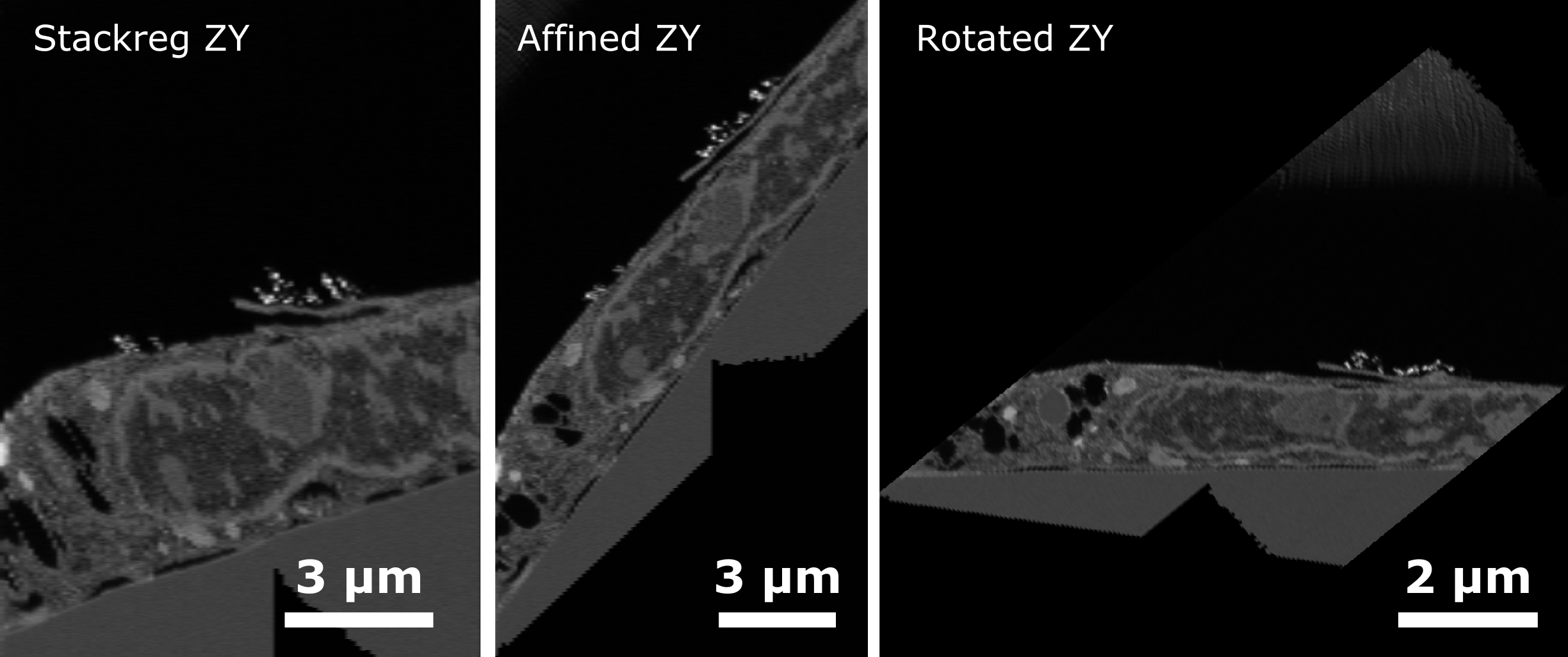


Figure 5 Sideviews of the obtained image stack showing the sequential processing operations’ effects. Left, the individual slices are aligned forming a fairly smooth image using stack-reg algorithm. Middle, then the substrate is corrected such as to annul the effects of automatic E-beam shifts in the Slice and view program, resulting in a 52 degree substrate. Right, finally the image stack is rotated 52 degrees to represent the sample on the flat substrate having been cut at an angle.

Particularly the non-tilted substrate required a large amount of computer memory, which meant that these samples had to be downscaled to 50%, thus decreasing the resolution of the images. In addition, some image ghosting and alignment errors occurred sometimes giving rise to ripples.


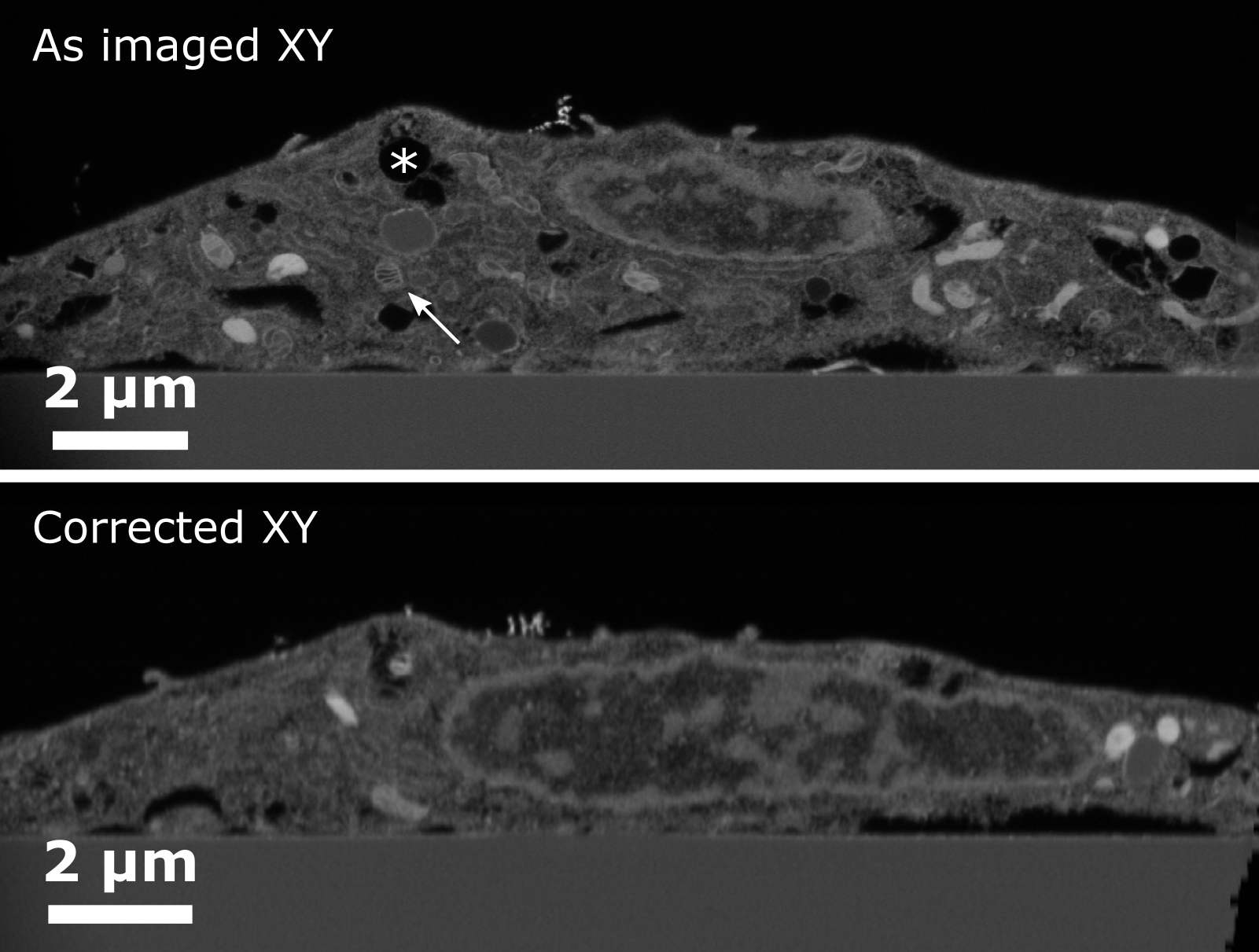


Figure 6 Top, high detail image of the as imaged cell y-scale corrected cell. The arrow indicates what looks similar to mitochondria. Also observed at * (and other) is what appears to be empty vesicles without staining inside the cytosol. Lower, a slice of the fully processed stack is shown. The corrected stack has less detail due to a decreased resolution limited by computer power, and the non-slanted milling approach where the quality here relies on the stackreg algorithm to limit misalignment between the multiple slices represented in this single image.

1. Thevenaz P, Ruttimann UE, Unser M (1998) A pyramid approach to subpixel registration based on intensity. IEEE Transactions on Image Processing 7: 27–41. doi:10.1109/83.650848.

2. Meijering EHW, Niessen WJ, Viergever MA (2001) Quantitative evaluation of convolution-based methods for medical image interpolation. Medical Image Analysis 5: 111–126. doi:10.1016/S1361-8415(00)00040-2.

1. Java based image processing and analysis program. Available from: http://rsbweb.nih.gov/ij/. [↑](#footnote-ref-1)
2. Stackreg plugin for imageJ. Available from: http://bigwww.epfl.ch/thevenaz/stackreg/ [↑](#footnote-ref-2)
3. TransformJ. Available from: http://www.imagescience.org/meijering/software/transformj/ [↑](#footnote-ref-3)
